# Genetic characterization and molecular epidemiological analysis of novel enterovirus EV-B80 in China

**DOI:** 10.1038/s41426-018-0196-9

**Published:** 2018-11-28

**Authors:** Zhenzhi Han, Yong Zhang, Keqiang Huang, Hui Cui, Mei Hong, Haishu Tang, Yang Song, Qian Yang, Shuangli Zhu, Dongmei Yan, Wenbo Xu

**Affiliations:** 1 WHO WPRO Regional Polio Reference Laboratory and National Health Commission Key Laboratory for Medical Virology, National Institute for Viral Disease Control and Prevention, Chinese Center for Disease Control and Prevention, Beijing, People’s Republic of China; 2 Xinjiang Uygur Autonomous Region Center for Disease Control and Prevention, Urumqi City, Xinjiang Uygur Autonomous Region, Beijing, People’s Republic of China; 3 Tibet Center for Disease Control and Prevention, Lhasa City, Tibet Autonomous Region, Beijing, People’s Republic of China; 4 Anhui University of Science and Technology, Anhui Province, People’s Republic of China

## Abstract

Enterovirus B80 (EV-B80) is a newly identified serotype belonging to the enterovirus B species. To date, only two full-length genomic sequences of EV-B80 are available in GenBank, and few studies on EV-B80 have been conducted in China or worldwide. More information and research on EV-B80 is needed to assess its genetic characteristics, phylogenetic relationships, and association with enteroviral diseases. In this study, we report the phylogenetic characteristics of three Xinjiang EV-B80 strains and one Tibet EV-B80 strain in China. The full-length genomic sequences of four strains show 78.8–79% nucleotide identity and 94–94.2% amino acid identity with the prototype of EV-B80, indicating a tendency for evolution. Based on a maximum likelihood phylogenetic tree based on the entire *VP1* region, three genotypes (A–C) were defined, revealing the possible origin of EV-B80 strains in the mainland of China. Recombination analysis revealed intraspecies recombinations in all four EV-B80 strains in nonstructural regions along with two recombination patterns. Due to the geographic factor, the coevolution of *EV-B* strains formed two different patterns of circulation. An antibody seroprevalence study against EV-B80 in two Xinjiang prefectures also showed that EV-B80 strains were widely prevalent in Xinjiang, China, compared to other studies on EV-B106 and EV-B89. All four EV-B80 strains are not temperature sensitive, showing a higher transmissibility in the population. In summary, this study reports the full-length genomic sequences of EV-B80 and provides valuable information on global EV-B80 molecular epidemiology.

## Introduction

The genus *Enterovirus* (EV), belonging to the family *Picornaviridae*, order Picornavirales, now contains 15 species assigned to enterovirus A–L as well as rhinovirus A–C^1^. Enterovirus A–D (EV-A, EV-B, EV-C, and EV-D) are species that can infect humans and consist of more than 100 serotypes, including poliovirus, coxsackievirus, echovirus, and some newly discovered enteroviruses. The genome is approximately 7.5 kb in length and contains a single long open reading frame (ORF) flanked by a 5′-untranslated region (UTR) and a 3′-UTR^2–4^. The polyprotein translated by the ORF ranges from 2138 to 2214 amino acids and can be cleaved into three polyprotein precursors, *P1*, *P2*, and *P3*, which are further cleaved into structural proteins *VP4*, *VP2*, *VP3*, and *VP1*, nonstructural proteins *2A*–*2* *C*, and nonstructural proteins *3A*–*3D*, respectively.

A series of infectious diseases caused by enteroviruses, including acute flaccid paralysis (AFP), hand, foot, and mouth disease (HFMD), myocarditis, aseptic meningitis, and others, were identified with the development of molecular typing technologies and other methods in recent years^5–7^. The *VP1* coding region of *Enterovirus* was used to identify the serotype because *VP1* is the most external and immunodominant region containing the most important specific antigenic neutralization sites among the capsid proteins^8–10^. The molecular typing method based on *VP1* coding region variation is now used to confirm the serotypes and has gradually replaced the traditional neutralization test^8,11–13^.

At present, EV-B comprises 63 serotypes, including coxsackievirus A9, coxsackievirus B group (serotypes 1–6), echovirus (serotypes 1–7, 9, 11–21, 24–27, 29–33), newly identified enteroviruses (serotypes 69, 73–75, 77–88, 93, 97–98, 100–101, 106–107, 110–113), and simian enterovirus SA5, that are usually associated with a range of diseases, especially AFP, aseptic meningitis and myocarditis. For example, coxsackievirus B3, usually linked to aseptic meningitis and myocarditis, can sometimes be fatal^14,15^. Repeated outbreaks of aseptic meningitis caused by echovirus 30 have been observed^16,17^. Some EV-B, such as coxsackievirus B5, have also been reported as the major pathogen in outbreaks of HFMD and aseptic meningitis^18,19^. Enterovirus B80 (EV-B80) is a newly identified serotype within EV-B. The prototype strain (CA67-10387/USA/1967) of EV-B80 was isolated in the USA in 1967 and was reported in 2007^5^. However, there are only two full-length genome sequences of EV-B80 in GenBank to date, including the prototype strain and one field strain (strain HZ01/SD/CHN/2004, isolated from an AFP case in Shandong Province of China in 2004)^5,20^. Further, only thirteen strains containing the entire *VP1* coding region sequence can be acquired from GenBank, excluding the two strains described above. Most reports of EV-B80 have involved sporadic detection and occasionally isolation. Few reports of EV-B80 have clearly illustrated the association with clinical diseases. Only one report clearly indicated that an EV-B80 strain (HZ01/SD/CHN/2004) was isolated from an acute flaccid paralysis (AFP) case in Shandong Province in China in 2004^20^. Therefore, little information on EV-B80 is available, and the etiological, epidemiological, and other features of EV-B80 remain unclear.

In this study, we determined the full-length genome sequences of four EV-B80 strains (strain *HT-LYKH203F/XJ/CHN/2011*, strain *HT-TSLH64F/XJ/CHN/2011*, strain *HTYT-XBBZH73F/XJ/CHN/2011*, and strain *KOUAN10067/XZ/CHN/2010*, hereafter referred to as *HT-LYKH203F*, *HT-TSLH64F*, *HTYT-XBBZH73F*, and *KOUAN10067*, respectively). Their genetic characteristics, phylogenetic relationships, recombination features, and other characteristics were investigated.

## Results

### Isolation and molecular typing of the four isolates

All four strains were cultured in human rhabdomyosarcoma (RD) cells and harvested when complete EV-like cytopathic effects (CPE) were observed. To determine the serotype of these four strains, reverse transcription polymerase chain reactions (RT-PCR) were performed using the previously described species-specific primers 490–492 and 491–493, covering the entire *VP1* coding region sequence^13^. The entire *VP1* coding region sequence was amplified. The EV Genotyping Tool (a BLAST server) results indicated that the query sequences showed 86% identity with known EV-B80 strains in GenBank and showed 73% identity with the EV-B80 prototype strain. The phylogenetic tree based on whole *VP1* and *P1* coding regions with EV-B prototypes confirmed the serotyping result. The four strains clustered with the EV-B80 prototype with a bootstrap value higher than 95% (Fig S3a-b), so all four strains were identified as EV-B80^21^.

### Full-length genomic characterization of the four EV-B80 strains

Full-length genome sequences of the four EV-B80 strains were identified. The results show that all strains are 7463–7464 nt in length, without a long poly(A) tail. The ORF of the four strains is 6618 nt in length, encoding a polypeptide of 2206 amino acids, with a 742 nt 5′-UTR and a 100-101 nt 3′-UTR. Alignment of the four EV-B80 strains in this study with the EV-B80 prototype (*UAS/CA67-10387*) indicated that all four strains have one nucleotide deletion at position 95 and two nucleotide insertions at positions 128 and 7373. Surprisingly, all 4 strains contain 36 nucleotide insertions at position 3322, similar to a previous report^20^, resulting in a 12 amino acid insertion in the *VP1* coding region (Fig S1). The Tibet strain *KOUAN10067* has two additional nucleotide deletions at positions 103 and 7390, whereas the three Xinjiang strains (*HT-LYKH203F*, *HT-TSLH64F*, *HTYT-XBBZH73F*) contain one additional nucleotide insertion at position 126.

The overall base compositions of the four strains are 28.4–28.8% A, 23.4% C, 24.4–24.6% G, and 23.4–23.6% T. The full-length genome nucleotide and amino acid similarities among the four strains were 83.9–99.1% and 96.8–99.5%, respectively. Moreover, the whole genome sequences of the four strains show 78.8–79% nucleotide identity and 94–94.2% amino acid identity with the prototype of EV-B80, whereas they show 81.8–83.3% nucleotide identity and 96.6–97.2% amino acid identity with the strain *HZ01/SD/CHN/2004* (data not shown). The four strains show higher similarity with strain *HZ01/SD/CHN/2004* in the *P1* region; in the *P2* and *P3* regions, the four strains show greater identity with some enterovirus prototype strains (such as prototype strain EV-B86, GenBank accession number AY843304, and prototype strain EV-B75, GenBank accession number AY556070) other than EV-B80, suggesting that recombination possibly occurs in these coding regions.

To assess the divergence among the four strains, nucleotide variation analysis was conducted using the strain *HT-LYKH203F* as a reference strain (Fig S2). The nucleotide base differences between *HT-LYKH203F* and *HT-TSLH64F* totaled 66 sites, whereas the nucleotide sequence differences between *HT-LYKH203F* and *HTYT-XBBZH73F* totaled 74 sites. The three Xinjiang EV-80 strains are highly similar to one another in the full-length genome. However, there are 1200 nucleotide base differences between the strains *HT-LYKH203F* and *KOUAN10067*. The nucleotide divergence of these two strains is spread across the entire genome except for a partial region of the 5′-UTR, indicating that the Tibet EV-B80 strain evolved in a different pattern from the three Xinjiang EV-B80 strains during their transmission.

### Phylogenetic analysis of the Chinese EV-B80 with other *EV-B* genomes

Phylogenetic trees based on the entire *VP1*, *P1*, *P2*, and *P3* coding region nucleotide sequences of the four EV-B80 strains with the prototypes of EV-B from GenBank were constructed (Fig S3). The *VP1* phylogenetic trees indicated that the four strains clustered with the prototype of *EV-B80* (*UAS/CA67-10387*) as expected, confirming the primary molecular typing results. Consistent with these results, the phylogenetic tree based on the *P1* coding region also showed that the four strains clustered with the prototype of EV-B80.

However, for the *P2* and *P3* coding region, the four strains did not cluster with the *EV-B80* prototype, suggesting that recombination occurred between EV-B80 and other serotypes (Fig S3). In the *P2* coding region tree, the Xinjiang EV-B80 strains were clustered together and shared the highest similarity with the prototype of *EV-B86* (*BAN00-10354*), whereas the Tibet EV-B80 strain shared the highest similarity with other *EV-B* prototypes. Similarly, in the *P3* coding region, the Xinjiang EV-B80 strains and the Tibet EV-B80 strain did not cluster together. These results suggest that the Xinjiang EV-B80 strain and the Tibet EV-B80 strain experienced different recombination patterns in the evolutionary process.

**Fig. 1:**
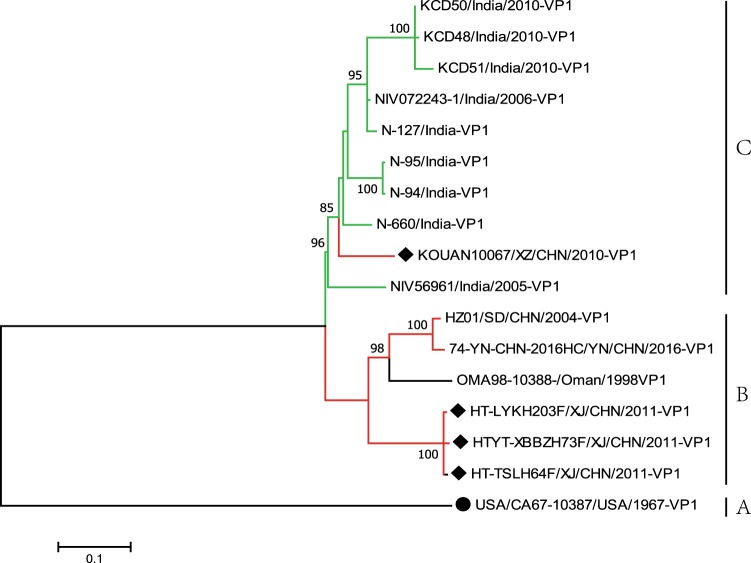
Maximum likelihood phylogenetic tree based on the entire *VP1* coding region sequences of EV-B80 available from GenBank. The four EV-B80 strains in this study are indicated by solid diamonds, and the prototype of EV-B80 is indicated by a solid circle. The branches are color-coded according to the location of sample collection (India = “green”, China = “red”). The scale bars indicate the substitutions per site per year. The numbers at the nodes indicate the bootstrap support for the node (percentage of 1000 bootstrap replicates)

To further study the evolutionary relationships and epidemic patterns of EV-B80 in the mainland of China, a maximum likelihood phylogenetic tree was constructed based on all EV-B80 *VP1* coding region sequences available in GenBank (Fig. 1). According to the usual practice, the prototype of EV-B80, which was isolated in the USA in 1967, formed a single lineage designated as genotype A. The three Xinjiang EV-B80 strains in this study were clustered together, whereas two other Chinese EV-B80 strains (HZ01/SD/CHN/2004 and HC/YN/CHN/2016) and an Oman strain (OMA98-10388-/Oman/1998) formed another cluster. All of the above six strains form a single lineage, designated as genotype B. Surprisingly, strain *KOUAN10067* was clustered with nine strains isolated from India and formed a single lineage, designated as genotype C (Fig. 1), indicating that strain *KOUAN10067* was possibly imported from India. The mean distance between the three genotypes is 19% and is larger than the mean distance within the three genotypes (13.3%), indicating the reliability of genotyping.

### Two recombination patterns of the four EV-B80 strains

**Fig. 2:**
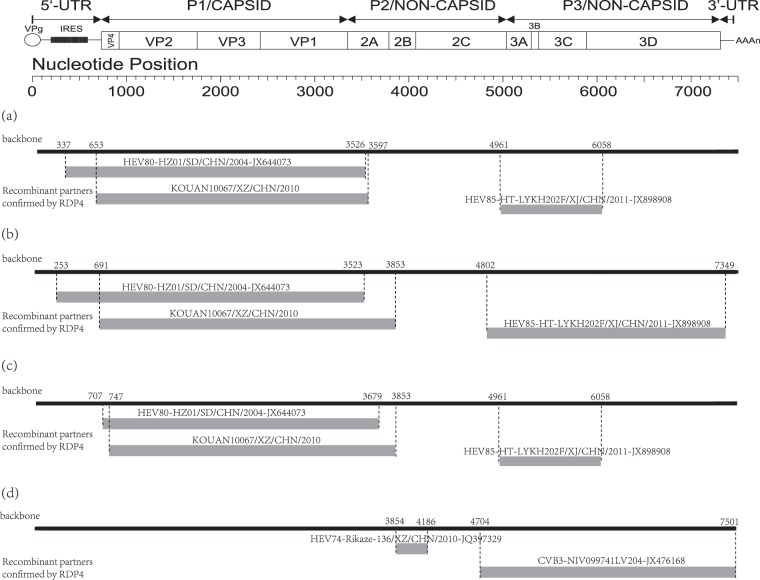
The genomic map (upper) and recombination events predicted for the four EV-B80 strains. The likely backbone and other genetic components were identified based on Table S2. The genome of EV-B80 is shown as a black block. Genetic components identified by RDP4 that were involved in recombination events are shown as light gray blocks. Likely breakpoint positions are shown above the genome. **a** Recombination events of *HT-LYKH203F*; **b** recombination events of *HT-TSLH64F*; **c** recombination events of *HTYT-XBBZH73F;* **d** recombination events of *KOUAN10067*

Through recombination analysis, we found clear evidence of intraspecies recombination. The recombination patterns of the three Xinjiang EV-B80 strains are very similar to one another. For example, in strain *HT-LYKH203F*, breakpoint positions were identified at positions 337–3526, 653–3597, and 4961–6058 (Table S2). Several EV-B strains, such as E-6, CV-B1, and EV-B85, were identified as the major and minor putative parents using RDP4 with six supported methods. Interestingly, the recombination patterns of strain *KOUAN10067* are completely different from those of the three Xinjiang EV-B80 strains. At position 3854-4186 in the alignment, several EV-B strains, such as EV-B74, E-27, and CV-B3, are considered as the major and minor putative parents with six supported methods (Fig. 2). It is obvious that these four strains underwent different recombination events in the evolutionary process.

Previous reports have shown that the Xinjiang EV-B85 strains (GenBank accession numbers JX898905– JX898909) and Xinjiang EV-B106 strains (KX171334–KX171337) underwent extensive genetic exchanges with other *EV-B* strains, including serotypes similar to themselves and other unknown serotype *EV-B* donor strains^22–24^. The results indicate that recombination between the Xinjiang EV-B85 strain (HTYT-ARL-AFP02F) and the Xinjiang EV-B106 strains (HTPS-QDH11F and KS-KSH28F) in the *3D* region was highly likely. Interestingly, recombination events also occurred between the EV-B85 strains described above and the three Xinjiang EV-B80 strains. In the *P3* region, recombination events among Xinjiang EV-B85, EV-B106, and the three Xinjiang EV-B80 strains were highly similar, illustrating that they possibly coevolved in the Xinjiang Uygur Autonomous Region before 2011 and that they possibly recombined with the same EV-B donor.

### Coevolution of the four EV-B80 strains with other *EV-B* serotypes

Detection of two recombination patterns of EV-B80 indicates that recombination is an approach for enterovirus evolution, which is also supported by other studies^25–31^. However, the exact donor sequences that offered the genetic fragment contributing to the recombination and circulation are usually difficult to identify. This also hindered us from analyzing the source of evolutionary process and epidemic characteristics^22,23^.

**Fig. 3:**
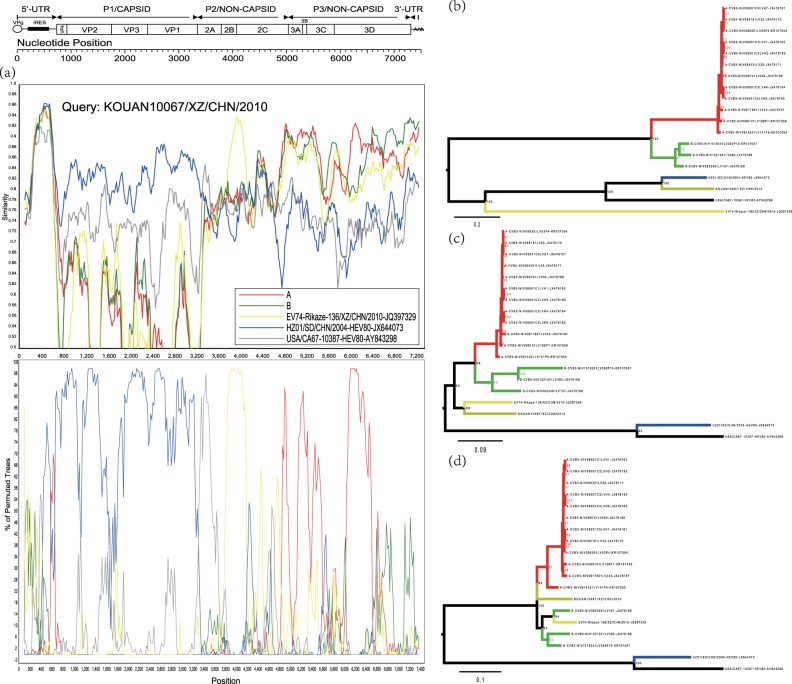
**a** Similarity and bootscanning analysis of strain *KOUAN10067* with *CV-B3* strains isolated from India, the prototype of *EV-B80*, a field EV-B74 strain (Rikaze-136/XZ/CHN/2010), and a field EV-B80 strain (*HZ01/SD/CHN/2004*). Strain *KOUAN10067* was used as a query sequence. **b** Midpoint-rooted maximum likelihood phylogenetic tree of isolates based on regions excluding *P2* and *P3* coding regions. **c** Midpoint-rooted maximum likelihood phylogenetic tree of isolates based on the *P2* coding region. **d** Midpoint-rooted maximum likelihood phylogenetic tree of isolates based on the *P3* coding region. All branches of the trees are colored according to the result of the bootscanning analysis except for strain *KOUAN10067* (dark yellow). The sequences analyzed in the recombination and phylogenetic trees were identical

To find the ancestral donor, multiple analyses, such as those including similarity plots, bootscanning analysis, and a midpoint-rooted maximum likelihood tree, were conducted. For the Tibet EV-B80 strain *KOUAN10067*, the results confirmed that it recombined with another Tibet EV-B74 strain, Rikaze-136/XZ/CHN/2010 (JQ397329), at position 3854-4816 (Figs. 3a, c and Table S2). It also experienced recombination events with strains of group A (see definition in Materials and methods) at position 4704-7501, as predicted by *RDP4* (Fig. 3d and Table S2).

**Fig. 4:**
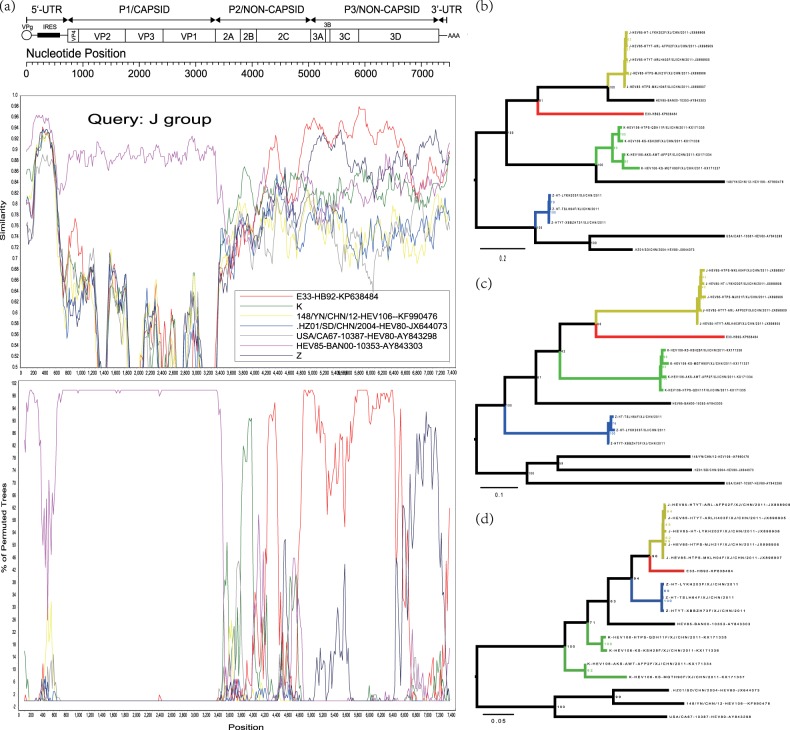
**a** Similarity and bootscanning analysis of EV-B85, EV-B106, and EV-B80 strains with potential parents. The J group was used as a query sequence. **b** Midpoint-rooted maximum likelihood phylogenetic tree of isolates was constructed based on regions excluding the *P2* and *P3* coding regions. **c** Midpoint-rooted maximum likelihood phylogenetic tree of isolates based on the *P2* coding region. **d** Midpoint-rooted maximum likelihood phylogenetic tree of isolates based on the *P3* coding region. All branches of the trees are colored according to the result of bootscanning analysis except for J group (dark yellow). The sequences analyzed in recombination and phylogenetic trees were identical

Recombination events were also assessed for the Xinjiang EV-B80 strains, EV-B106 strains, and EV-B85 strains using similarity plots and bootscanning analysis. When the EV-B80 strains were used as query strains for analysis, the signal of recombination among EV-B80, EV-B85, and EV-B106 was strong enough that the ancestral “donor” could not be identified. When the EV-B85 strains were used as query strains, the signal of the ancestral “donor” could be clearly identified. To definitively illustrate the recombination, three groups were defined based on the enterovirus serotype. The J group (dark yellow line), consisting of five Xinjiang EV-B85 strains, was used as the query sequence; the K group (green line) was composed of four Xinjiang EV-B106 strains; and the Z group (blue line) consisted of the three Xinjiang EV-B80 strains in this study. In the 5′-UTR and *P1* region, the J group of the EV-B85 strains shared the highest similarity with the EV-B85 prototype strain (BAN00-10353-AY843303) (Fig. 4a). In the *2B* coding region, the query strain possibly recombined with the K group (Xinjiang EV-B106). However, in the *2C-3D* coding region, the query strains shared the highest identity with the E-33 strain (HB92-KP638484, isolated in China in 2014), with a high support value. The results were also supported by the maximum likelihood phylogenetic trees (Figs. 4b, c). In the *3D* coding region, the query strains recombined with the Z group (EV-B80) with a higher probability. In the *3A-3D* coding region, the E-33 strain HB92-KP638484 was a potential donor supported by the maximum likelihood phylogenetic analysis (Fig. 4d). This suggested that the cocirculation and multiple recombination of EV-B strains occurred in Xinjiang before 2011.

### Seroprevalence of EV-B80 in Xinjiang

In total, 48 serum samples were collected from newborns to 4-year-old children from Xinjiang, including 24 serum samples collected from Kashgar Prefecture and 24 samples collected from Hotan Prefecture. Among the 48 serum samples, 24 were seropositive samples (>1:8) for EV-B80, with a total positive rate of 50% and geometric mean titers (GMTs) of 1:15.77. The composition ratios for the EV-B80 neutralization antibody titers of <1:8, 1:8–1:64, and >1:64 were 18.75, 68.75, and 12.5%, respectively. However, compared with the sero-epidemiological studies of other EVs in China, such as EV-A71 and CV-A16, the positive rates of the EV-B80 neutralization antibody and GMTs were slightly lower than those of EV-A71 and CV-A16 in the same age group (0–4 years old)^32^.

In Kashgar Prefecture, the positive rates of neutralization antibody and GMTs were 58.3% and 1:20.16, respectively. In Hotan Prefecture, the positive rates of neutralization antibody and GMTs were 41.7% and 1:12.34, respectively. This indicated that small-scale epidemics of EV-B80 occurred in these two regions compared with other reports on EV-106 and EV-89^24,33^.

### Xinjiang EV-B80 strains are not temperature sensitive

**Fig. 5:**
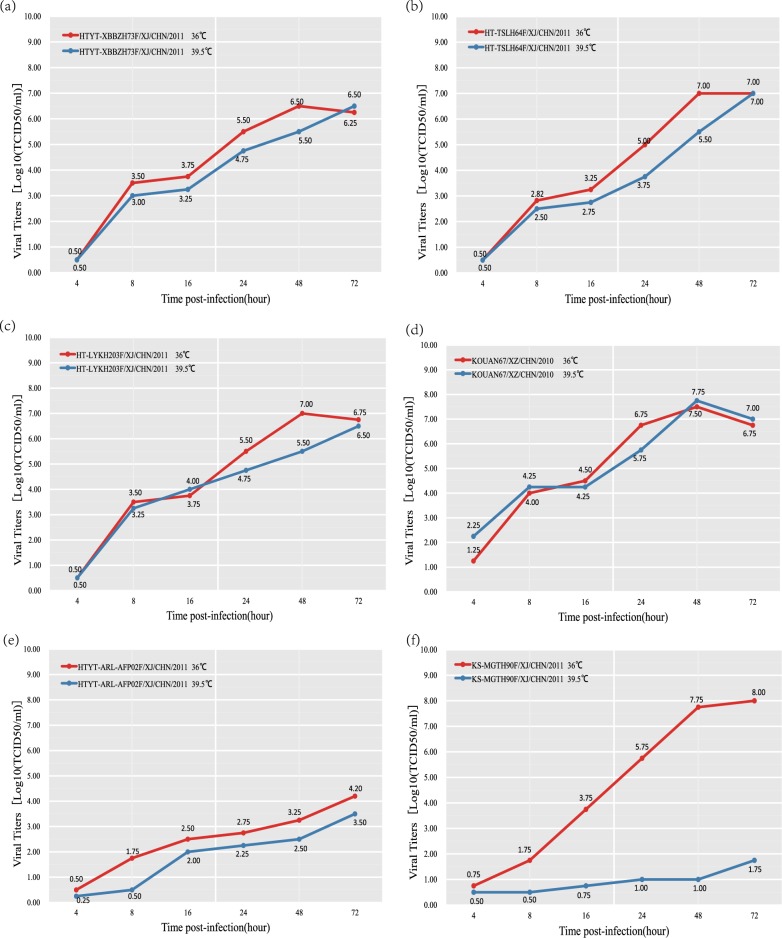
Temperature sensitivity test curves of the four EV-B80 strains. Blue and red lines represent the growth trends of the viruses on RD cells at 36 and 39.5 °C, respectively. The Xinjiang EV-B85 strain (HTYT-ARL-AFP02F/XJ/CHN/2011, showing nontemperature sensitivity) and the EV-B106 strain (KS-MGTH90F/XJ/CHN/2011, showing temperature sensitivity) were used as experimental controls. **a** strain *HTYT-XBBZH73F*; **b** strain *HT-TSLH64F*; **c** strain *HT-LYKH203F*; **d** strain *KOUAN10067* **e** strain *HTYT-ARL-AFP02F* (*EV-B85*); **f** strain *KS-MGTH90F* (*EV-B106*)

All four EV-B80 strains were compared with one another with respect to their replication capacity at an elevated temperature (39.5 °C). The results indicated that all four isolates are not temperature sensitive, with a titer reduction lower than 2 logarithms at 36 and 39.5 °C (Fig. 5). These four EV-B80 strains show higher tolerance to temperature compared to the EV-B106 strains, which show temperature sensitivity. Although the four EV-B80 strains showed different capacities of propagation at postinfection time points, their growth curves remained similar.

## Discussion

The isolation of enteroviruses in cell culture followed by serotyping based on a neutralization assay has long been regarded as the gold standard for diagnosing enteroviral infections. However, the traditional neutralization assay is labor intensive and time consuming, and it is impractical in outbreak situations, where rapid and accurate diagnosis at an early stage of enteroviral infection is needed. Thus, the “molecular serotyping” method has gradually replaced the neutralization assay, and it is now the major EV serotyping gold standard used in the laboratory. When molecular typing methods became the gold standard for enterovirus detection, an increasing number of enteroviruses were found^8^. EV-B80 was a newly identified enterovirus serotype in 2007^5^. However, few studies on EV-B80 have been reported globally since then. In this study, four EV-B80 strains, including three Xinjiang strains and one Tibet strain, were isolated and characterized. Compared with the EV-B80 prototype strain, the four Chinese EV-B80 strains in this study show significantly different characteristics. Surprisingly, all four strains contain thirty-six nucleotide insertions at position 3322 in the *VP1* region, similar to other EV-B80 strains (such as strain OMA98-10388) available in GenBank^20^. The *VP1* coding region is known to be very important, as it encodes the antigenic neutralization sites of enteroviruses. A twelve amino acid insertion in the *VP1* protein might thus have a severe influence on the virus phenotype.

Previous studies showed that EV-B80 strains were occasionally detected during different time periods in different geographic regions^20,34–36^. In this study, we further divided all of the EV-B80 strains into three groups, genotypes A, B, and C. Interestingly, an Oman strain (OMA98-10388/Oman/1998) clustered with most of the Chinese strains, indicating that Oman might be the possible origin of the EV-B80 strains in China. In contrast, the strains isolated in India clustered together with the Tibet strain *KOUAN10067*, indicating that the strain *KOUAN10067* was more likely to be imported from India (Fig. 1).

The cocirculation or coevolution of some novel serotypes of enteroviruses apparently occurred in Xinjiang, China, before 2011; another study also showed similar insights with enterovirus C (*EV-C*)^27^. Multiple reports on the cocirculation of enteroviruses also showed that recombination is the driving force and leads to the emergence of lineages with altered properties^37,38^. Frequent recombination and mutations in enteroviruses have been recognized as the main mechanisms for their evolution, enabling them to rapidly respond and adapt to new environments. The accumulation of interspecies and intraspecies recombination events could be a strong driver of the emergence and disappearance of certain enterovirus serotypes. Many reports show that intraspecies recombination events have occurred frequently and that *EV-B* is more susceptible to recombination^28,39^. The present results also confirmed that strains of EV-B80, a member of *EV-B*, underwent frequent intraspecies recombination during its global circulation.

The results show that Chinese EV-B80 had two recombination patterns. The first recombination pattern occurred among EV-B80, EV-B106, EV-B85, and other donor strains in Xinjiang, China. The other pattern showed different recombination events among EV-B74 strains, CV-B3 strains, and other donor strains. It is widely accepted that two different enteroviruses can only exchange genetic materials in case of coinfection in the same host cell. Due to geographic factors, enteroviruses circulated in populations in different locations that were possibly infected by different dominating enteroviruses, resulting in different opportunities of gene exchange. It has thus been interpreted that EV-B80 strains underwent different recombination patterns in different locations.

To investigate the epidemic intensity of EV-B80 in Xinjiang, China, seroepidemiological surveys were conducted. The results of positive seroprevalence rates and GMTs showed that EV-B80 strains prevailed widely and were spread in Xinjiang compared to EV-B106 and EV-B89^24,33^. However, the extent of transmission and exposure of the population to the novel EV-B80 serotypes was highly limited compared to EV-A71 and CV-A16^32^, which have caused large outbreaks in China since 2008^10,40^.

All four EV-B80 isolates showed a non-temperature-sensitive phenotype, which is usually an indicator of high virulence or transmissibility^41,42^. Indeed, the seroepidemiological survey confirmed the reality that EV-B80 strains are widely spread in Xinjiang compared to other *EV-B* strains, such as EV-B89 and EV-B106^24,33^. This is consistent with the fact that all four isolates are not temperature sensitive strains, which may indicate higher transmissibility. However, this still needs to be confirmed by strengthening virological surveillance to prevent potential outbreaks caused by EV-B80.

In conclusion, we reported the full-length genome sequences of four EV-B80 strains during AFP case surveillance in Xinjiang and Tibet, supporting global polio eradication. Isolates from these two regions showed high genetic diversity and different intertypic recombination patterns in the nonstructural coding region. The high divergence among EV-B80 strains indicated that these viruses have circulated in the environment for many years and have undergone separate evolution. More enterovirus surveillance studies are needed to improve our knowledge and evaluate the association between enterovirus and enterovirus-related diseases, such as AFP^43^. The current surveillance and basic research efforts must be strengthened to completely understand viral pathogenesis and develop effective medical countermeasures^44^. This report provides valuable information about EV-B80 isolates and expands the number of entire genome sequences of EV-B80 in the GenBank database to help prevent enterovirus-related diseases.

## Materials and methods

### Sample collection

The only materials used were stool samples collected from four healthy persons for public health purposes. Moreover, written informed consent for the use of their clinical samples was acquired from the parents of all children involved in this study. This research was supported by the Ethics Review Committee of the National Institute for Viral Disease Control and Prevention, Chinese Center for Disease Control and Prevention. All experimental protocols were approved by the National Institute for Viral Disease Control and Prevention, and the methods were carried out in accordance with the approved guidelines.

The four strains (*HT-LYKH203F*, *HT-TSLH64F*, *HTYT-XBBZH73F*, *KOUAN10067*) were isolated from stool specimens of four healthy persons. The former three were isolated from fecal samples of healthy people, collected in Hotan Prefecture of the Xinjiang Uygur Autonomous Region of China in 2011, and the last one was isolated from a fecal sample of a healthy person in the Tibet Autonomous Region of China in 2010 for the aim of poliovirus eradication by the World Health Organization (WHO).

In the seroprevalence study for EV-B80 antibodies, 48 serum samples were collected from 48 healthy children (≤5 years of age) in 2013, with informed parental consent, by the Xinjiang Center for Disease Control and Prevention, including 24 samples from Hotan Prefecture and 24 samples from Kashgar Prefecture. None of the children had any signs of disease at the time of sample collection.

### Viral isolation and molecular typing

Stool specimens from four healthy children were processed according to standard procedures^45^ and inoculated into the RD cell line for viral isolation. The cell lines were provided by the WHO Global Poliovirus Specialized Laboratory in the USA and were originally purchased from the American Type Culture Collection (Manassas, VA, USA). After complete EV-like CPE were observed, we harvested the infected cell cultures.

Viral RNA was extracted from the cell culture using a QIAamp Viral RNA Mini Kit (Qiagen, Hilden, Germany). RT-PCR was performed to amplify the partial *VP1* coding region using the PrimeScript One Step RT-PCR Kit Ver.2 (TaKaRa, Dalian, China) with primers 490 and 492^13^. The PCR products were purified using the QIAquick PCR purification kit (Qiagen, Hilden, Germany). An ABI 3130 Genetic Analyzer (Applied Biosystems, Foster City, CA, USA) was then used to sequence in both directions and at least once from each strand. The EV Genotyping Tool (a BLAST server) and maximum likelihood phylogenetic trees based on two regions (entire *VP1* region and entire *P1* region) were used for enterovirus serotyping^21^.

### Full-length genome sequencing of the four EV-B80 strains

The full-length genome sequences of the virus were amplified by the “primer-walking” strategy, which was used to close the gaps as necessary^7^. Briefly, overlapping fragments representing whole genomes were amplified by *RT-PCR* using specific primers (Table S3). The RT-PCR products were purified for sequencing using the QIAquick Gel extraction kit (Qiagen, Hilden, Germany), and the amplicons were sequenced on an ABI 3130 Genetic Analyzer (Applied Biosystems, Foster City, CA, USA) as described above. The 5′ end of the genome was amplified based on the manufacturer’s instructions with the 5′-Full RACE Kit (Takara, Shiga, Japan). The 3′ end of the genome was amplified using an oligo-dT primer (7500 A) previously reported in another study^46^.

### Phylogenetic and recombination analysis

Full-length genomic sequences of the four strains and deduced amino acid sequences were aligned with the EV-B prototype strains using the ClustalW algorithm implemented in MEGA7^47^. A nucleotide identity matrix was generated using BioEdit^48^. Differences across the entire genomic sequences were also analyzed using the Seqcombo package implemented in R to identify the variant sites^49^. Maximum likelihood trees were constructed using the GTR + I + G model as suggested by jModelTest2^50^ and were implemented in MEGA7 with 1000 bootstrap replicates. Maximum likelihood trees were also constructed using RAxML (v8.2.10) to verify the best topology of trees^51^.

The Recombinant Detection Program (RDP4, v4.46) was used to screen for recombination signals in our set of whole genome sequences using seven methods (RDP, GENECONV, MaxChi, Bootscan, Chimaera, SiScan and 3Seq)^52^. Briefly, the *P2* and *P3* coding region sequences of the four strains were analyzed using the BLAST server to compare their identity with sequences from GenBank. Sequences with a similarity higher than 85% were considered potential parents of the four strains and were downloaded from GenBank. Phylogenetic incongruence between different regions with *P* values less than 0.05 was considered strong evidence for recombination. We only considered recombination events that were identified by at least three methods. To confirm these putative recombination events, we utilized a smaller data set including recombinant and parental strains for multiple screenings. Due to a higher similarity of *P3* coding region with CV-B3 strains from GenBank, these strains were downloaded and analyzed for screening recombination. Fifteen *CV-B3* strains isolated from India were grouped according to the whole genome nucleotide-sequence identity, and sequences with identities higher than 90% were clustered into one group. Here, to clearly distinguish the recombination events, two groups of CV-B3 strains isolated from India were defined. Group A (red line) consists of 12 CV-B3 sequences (GenBank accession numbers: *JX476161*–*JX476165*, *JX476167*–*JX476168, JX476170*–*JX476171, KR107054*–*KR107056*). Group B (green line) comprises three CV-B3 sequences (GenBank accession numbers: *JX476166, JX476169*, and *KR107057*). The SimPlot program (version 3.5.1) was used for similarity plots and bootscanning analysis, with a 200-nucleotide window moving in 20-nucleotide steps^53^. Recombination breakpoints were inferred according to the distribution of informative sites, which supported two incongruent tree topologies that maximized the chi-square (χ^2^) sum^54^.

### Test of neutralization

After a plaques experiment was performed to purify the viral mixture culture, a neutralization test was performed to detect neutralizing antibodies using human RD cell lines as described previously^55^. EV-B80 Strain *HT-TSLH64F* was chosen as the attack virus in the neutralizing test because it showed the highest titer among the four EV-B80 strains. In total, 48 serum samples were inactivated at 56 °C for 30 min, with sample dilutions from 1:4 to 1:1024 to be assayed. The mixture of virus samples consisting of a culture infection dose (CCID_50_) of 100 (50 μL), human RD cell lines (100 μL) and serum dilution (50 μL) were then incubated at 36 °C in a 5% CO_2_ incubator. With observation for 7 days, the highest dilution of serum that protected 50% of the cultures was recorded based on EV-like CPE. If the neutralization antibody titer was observed at a dilution higher than 1:8, a serum sample was considered positive and the GMT was subsequently calculated.

### Assay of temperature sensitivity

The temperature sensitivity of the four plaque-purified EV-B80 strains and two selected control strains (HTYT-ARL-AFP02F/XJ/CHN/2011, showing nontemperature sensitivity and KS-MGTH90F/XJ/CHN/2011, showing temperature sensitivity) were assayed on monolayer RD cells in 24-well plates^41^. The 24-well plates were inoculated with 50 μL undiluted virus stocks. Two different incubators were used: one incubator was adjusted to 36 °C as the optimal temperature for virus propagation, and another was adjusted to 39.5 °C as the supraoptimal temperature for virus propagation. After adsorption at 36 °C or at 39.5 °C for 1 h, the unadsorbed virus inoculum was removed and 100 μL of maintenance medium was added to each well. The plates were continually incubated at 36 or at 39.5 °C and harvested at 6 time points postinfection (4, 8, 16, 24, 48, and 72 h) in succession. The CCID_50_ was calculated by the end-point dilution method on monolayer RD cells in 96-well plates at 36 °C. Virus isolates showing more than 2-logarithm reduction in titer at different temperatures were considered temperature-sensitive^23,41,42,55^.

## Electronic supplementary material

supplemental materials

## Data Availability

Whole genome nucleotide sequences for the four strains determined in this study have been deposited in the GenBank nucleotide sequence database under accession numbers MH614922-MH614925.
