# Supplementary material for: Genetic characterization and molecular epidemiological analysis of novel enterovirus EV-B80 in China
Source: Emerg Microbes Infect. 2018 Nov 28;7:193. doi: 10.1038/s41426-018-0196-9 (PMC6258725; doi:10.1038/s41426-018-0196-9)
Supplement: Supplementary file 1 — supplemental materials [file 41426_2018_196_MOESM1_ESM.pdf]

Table S1. Pairwise nucleotide and amino acid sequence identities among the four EV-B80 strains, prototype of EV-B80(USA/CA67-10387/USA/1967), HZ01/SD/CHN/2004 strain, and other prototypes of the EV-B species. The gray shading represent amino acids identity.

	Nucleotide identity(%) [Amino acid identity(%)]											
Region	HT-LYKH203F/XJ/CHN/2011			HT-TSLH64F/XJ/CHN/2011			HTYT-XBBZH73F/XJ/CHN/2011			KOUAN10067/XZ/CHN/2010		
	HEV80-USA/CA67/10387	HEV80-HZ01/SD/CHN/2004	Prototype of other EV-B	HEV80-USA/CA67/10387	HEV80-HZ01/SD/CHN/2004	Prototype of other EV-B	HEV80-USA/CA67/10387	HEV80-HZ01/SD/CHN/2004	Prototype of other EV-B	HEV80-USA/CA67/10387	HEV80-HZ01/SD/CHN/2004	Prototype of other EV-B
5'-UTR	85.3	87.3	65.6-88.6	85.5	87.4	65.7-88.8	85.7	87.6	65.3-88.8	84.5	87.3	65.7-90.1
VP4	78.7	85.5	63.2-78.7	79.2	85	63.7-79.2	79.7	84.5	63.2-79.7	76.3	81.6	65.2-78.2
	(84)	(97.1)	(68.1-84)	(84)	(97.1)	(68.1-84)	(84)	(97.1)	(68.1-84)	(85.5)	(97.1)	(71-85.5)
VP2	78.5	87.7	66.4-78.5	78.6	87.9	66.6-78.6	78.7	87.9	66.4-78.7	79	84.2	66.2-79
	(94.6)	(98.8)	(74-94.6)	(94.6)	(98.8)	(74-94.6)	(95)	(98.4)	(74-95)	(94.6)	(97.7)	(74-94.6)
VP3	77.2	86.7	63.1-77.1	76.5	86.8	63.4-76.4	76.9	86.7	63.8-76.8	77.8	82.9	63.6-77.7
	(94.9)	(99.1)	(69-94.9)	(94.5)	(99.5)	(69-94.5)	(94.9)	(99.1)	(69-94.9)	(94.1)	(98.3)	(68.6-94.1)
VP1	73.1	85.6	53.8-73.1	73	85.4	53.5-73	72.6	85.4	53.7-72.6	72.9	84.8	53.1-72.9
	(86.7)	(96)	(52.9-87.1)	(86.7)	(96)	(52.9-87.1)	(86.1)	(95.7)	(52.2-86.4)	(87.1)	(95.7)	(52.5-86.7)
2A	78	82.4	63.1-81.5	77.7	83.1	63.1-81.3	78.4	82.6	62.8-81.1	81.7	79.7	64.4-81.7
	(92.6)	(94.6)	(62.6-94.6)	(92.6)	(94)	(62.6-94)	(93.3)	(94.6)	(62.6-94.6)	(94)	(95.3)	(64.6-97.3)
2B	82.1	80.4	70.3-86.5	81.4	81.4	70-86.1	81.8	80.8	70-86.1	80.8	77.4	67.6-83.5
	(94.9)	(94.9)	(77.7-98.9)	(95.9)	(95.9)	(77.7-100)	(95.9)	(95.9)	(77.7-100)	(96.9)	(92.9)	(74.7-98.9)
2C	81.6	80.7	75-86	81.9	81.1	75-85.8	81.7	80.5	75.2-85.6	81.3	80.5	74-84.2

	(98.7)	(98.4)	(87.8-99.6)	(98.7)	(98.4)	(87.8-99.6)	(98.4)	(98.1)	(87.8-99.3)	(96.9)	(97.2)	(88.1-98.4)
3A	79	80.5	70.7-83.8	79.4	80.1	70.7-83.5	79.4	80.1	70.7-83.5	78.2	81.6	69.6-84.6
	(95.5)	(98.8)	(84.2-98.8)	(95.5)	(98.8)	(84.2-98.8)	(95.5)	(98.8)	(84.2-98.8)	(96.6)	(98.8)	(84.2-1)
3B	74.2	72.7	71.2-84.8	74.2	72.7	71.2-86.3	74.2	72.7	71.2-86.3	77.2	72.7	71.2-86.3
	(100)	(100)	(81.8-100)	(100)	(100)	(81.8-100)	(100)	(100)	(81.8-100)	(95.4)	(95.4)	(77.2-95.4)
3C	77	78.3	71.5-85.9	77	78.3	71.9-85.6	77.4	78.3	71.7-85.7	76.5	78.1	70.8-84.5
	(95)	(96.1)	(80.8-99.4)	(94.5)	(95.6)	(81.4-98.9)	(95)	(96.1)	(80.8-99.4)	(93.9)	(96.1)	(80.8-98.3)
3D	78.4	80.3	70.4-86.9	78.3	80.3	70.4-86.9	78.5	80.2	70.2-86.7	78.3	79.2	70.2-88.9
	(96.3)	(96.7)	(82.2-98.4)	(96.3)	(96.7)	(82.2-98.4)	(96.5)	(96.7)	(82-98.7)	(96.3)	(96.1)	(81.3-98.4)
3'-UTR	84.1	84.3	59.4-93.2	84.1	84.3	59.4-93.2	84.1	84.3	59.4-93.2	84.1	83.3	60.3-92.2

Table S2. The results of recombination analysis implemented by RDP4 packages.

Recombinant	Breakpoint position[ψ]		Region[δ]	Major parent	Minor parent	Methods*						
	Beginning breakpoint	Ending breakpoint				RDP	Geneconv	BootScan	MaxChi	Chimaera	SiScan	3Seq
HT-LYKH203F/XJ/CHN/2011	337(330)	3526(3482)	5'UTR,VP4,VP2,VP3,VP1,2A	E6-EV6-10887-99/Russia-AY896760	HEV80-HZ01/SD/CHN/2004-JX644073	1.31×10^{-3}	NA	NA	4.196×10^{-8}	1.509×10^{-7}	1.781×10^{-53}	1.517×10^{-8}
	653(643)	3597(3553)	5'UTR,VP4,VP2,VP3,VP1,2A	CVB1-_CVB1-Chi07-KJ849619	KOUAN10067/XZ/CHN/2010	7.924×10^{-5}	1.649×10^{-4}	NA	4.222×10^{-6}	5.476×10^{-5}	9.665×10^{-45}	5.506×10^{-13}
	4961(4917)	6058(6014)	2C,3A,3B,3C,3D	KOUAN10067/XZ/CHN/2010	HEV85-HT-LYKH202F/XJ/CHN/2011-JX898908	8.494×10^{-16}	NA	9.391×10^{-21}	2.126×10^{-15}	5.621×10^{-8}	2.073×10^{-16}	1.101×10^{-12}
HT-TSLH64F/XJ/CHN/2011	253(246)	3523(3479)	5'UTR,VP4,VP2,VP3,VP1,2A	E6-EV6-10887-99/Russia-AY896760	HEV80-HZ01/SD/CHN/2004-JX644073	1.31×10^{-3}	NA	NA	4.196×10^{-8}	1.509×10^{-7}	1.781×10^{-53}	1.517×10^{-8}
	691(681)	3853(3809)	5'UTR,VP4,VP2,VP3,VP1,2A	CVB1-_CVB1-Chi07-KJ849619	KOUAN10067/XZ/CHN/2010	7.924×10^{-5}	1.649×10^{-4}	NA	4.222×10^{-6}	5.476×10^{-5}	9.665×10^{-45}	5.506×10^{-13}

	4802(4758)	7349(7305)	2C,3A,3B,3C,3D	KOUAN10067/XZ/CHN/2010	HEV85-HT-LYKH202F/XJ/CHN/2011-JX898908	8.494×10^{-16}	NA	9.391×10^{-21}	2.126×10^{-15}	5.621×10^{-8}	2.073×10^{-16}	1.101×10^{-12}
HTYT-XBBZH73F/XJ/CHN/2011	707(697)	3679(3635)	5'UTR,VP4,VP2,VP3,VP1,2A	E6-EV6-10887-99/Russia-AY896760	HEV80-HZ01/SD/CHN/2004-JX644073	1.31×10^{-3}	NA	NA	4.196×10^{-8}	1.509×10^{-7}	1.781×10^{-53}	1.517×10^{-8}
	747(735)	3853(3809)	5'UTR,VP4,VP2,VP3,VP1,2A	CVB1-_CVB1-Chi07-KJ849619	KOUAN10067/XZ/CHN/2010	7.924×10^{-5}	1.649×10^{-4}	NA	4.222×10^{-6}	5.476×10^{-5}	9.665×10^{-45}	5.506×10^{-13}
	4961(4917)	6058(6014)	2C,3A,3B,3C,3D	KOUAN10067/XZ/CHN/2010	HEV85-HT-LYKH202F/XJ/CHN/2011-JX898908	8.494×10^{-16}	NA	9.391×10^{-21}	2.126×10^{-15}	5.621×10^{-8}	2.073×10^{-16}	1.101×10^{-12}
KOUAN10067/XZ/CHN/2010	3854(3810)	4186(4142)	2B,2C	HEV80-HZ01/SD/CHN/2004-JX644073	HEV74-Rikaze-136/XZ/CHN/2010-JQ397329	2.385×10^{-15}	5.16×10^{-9}	3.946×10^{-15}	2.057×10^{-3}	4.586×10^{-2}	2.595×10^{-6}	NA
	4704(4660)	7501(7454)	2C,3A,3B,3C,3D,3'UTR	E27-Bacon-AY302551	CVB3-NIV099741LV204-JX476168	3.805×10^{-15}	NA	2.585×10^{-21}	7.998×10^{-4}	2.11×10^{-11}	1.033×10^{-23}	5.295×10^{-5}

Ψ represent the position of breakpoint position in alignment(the numbers within brackets represent breakpoint position without gaps);

* represent the P-value given by RDP4 packages;

δ represent the genomic structure of enterovirus which was related to recombination.

Table S3. RT-PCR and sequencing primer.

Primer	Nucleotide position(nt)	Sequence(5’-3’)	Orientation	Reference
0001S48		GGGGACAAGTTTGTACAAAAAAGCAGGCTTTAAAACAGCTCTGGGGTT	Forward	43
5’RACE-inner-primer-EV541	541-564	AACACGGACACCCAAAGTAGTCGG	Reverse	This study
5’RACE-outer-primer-EV883	883-905	GAGTTGGAGGCTGCATCTTTGTA	Reverse	This study
EVP4	541–560	CTACTTTGGGTGTCCGTGTT	Forward	42
OL68-1	1178–1197	GGTAAYTTCCACCACCANCC	Reverse	42
EV846	846-865	ATGCAGCCTCCAACTCCTCC	Forward	This study
EV1743	1743-1763	TACTCCCTGGTGTGTTCAACA	Reverse	This study
EV2216	2216-2235	CACAGTACGCAGCTGGATTG	Reverse	This study
EV2216-2	2216-2235	CACAATACACAGCTGGATTG	Reverse	This study
EV1624	1624-1643	CCCATTCGCACCATTGAATT	Forward	This study
EV2723	2723-2742	TCTCTCGTGGTGATTTCCCA	Reverse	This study
EV1331	1331-1352	CCTGAAGCTGAGATGGGATGT	Forward	This study
490	2226–2248	TGIGTIYTITGYRTICCITGGAT	Forward	This study
491	2883–2902	ATGTAYRTICCICCIGGNGG	Forward	This study
492	2953–2934	GGRTTIGTIGWYTGCCA	Reverse	This study
493	3641–3622	TCNACIANICCIGGICCYTC	Reverse	This study
EV2979	2979-2998	CCACCGCGCATGTCCATCCC	Forward	This study
EV4525	4525-4545	GCTAGTGATCGCCCGATCAAA	Reverse	This study
EV3460	3460-3479	GGACTAYAACAGGGATCTCT	Forward	This study

EV4559	4559-4578	GGCAAGGAATACACCGAGCT	Reverse	This study
EV4376	4376-4396	GCACCCCTATATGCTGCTGAG	Forward	This study
EV4297	4297-4317	GCAAAGTGCACCCTCGCAAAG	Forward	This study
EV5374	5374-5394	TTGGGCTTTTGATTGGGCATC	Reverse	This study
EV5186	5186-5206	GAAGCAGTGAGGGAGTACTGT	Forward	This study
EV6192	6192-6212	GGTCCACTGCCTCCATCATAT	Reverse	This study
EV6059	6059-6079	GTGTTCCACCAGGTGTTTGAG	Forward	This study
EV6816-2	6816-6835	ATTTTGTGAGGGGCGGAATG	Forward	This study
EV6816-3	6816-6837	ACTTTGTAAGAGGAGGAATGCC	Forward	This study
EV7098	7098-7118	CTGCCCTGAAGTACCTCTTCA	Reverse	This study
7500A		GGGGACCACTTTGTACAAGAAAGCTGGG$(T)_{24}$	Reverse	43

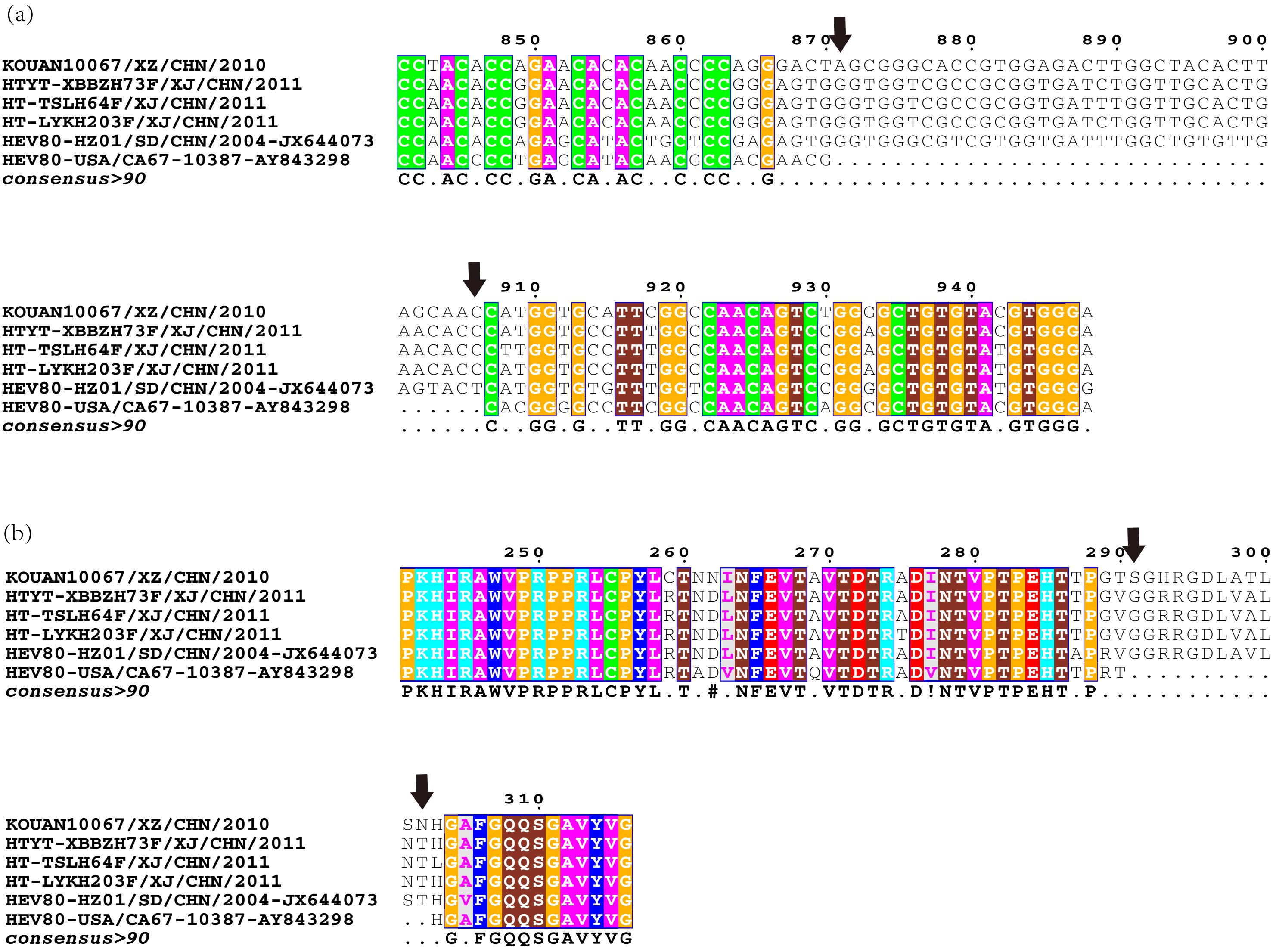

Fig S1. (a)Nucleotide sequence alignment of the partial VP1 coding region with the EV-B80 prototype strain and a field strain (HZ01/SD/CHN/2004). (b)Amino acid sequence alignment of partial VP1 region. The black arrow between forward and downstream of sequences show nucleotide insertion position and amino acid insertion position, respectively. The black dot represent the deletion of nucleotides or amino acids. The bottom of "consensus>90" show that the identify of nucleotides and amino acids higher than 90% were shown. The positions of sequences were numbered according to the entire VP1 coding region.

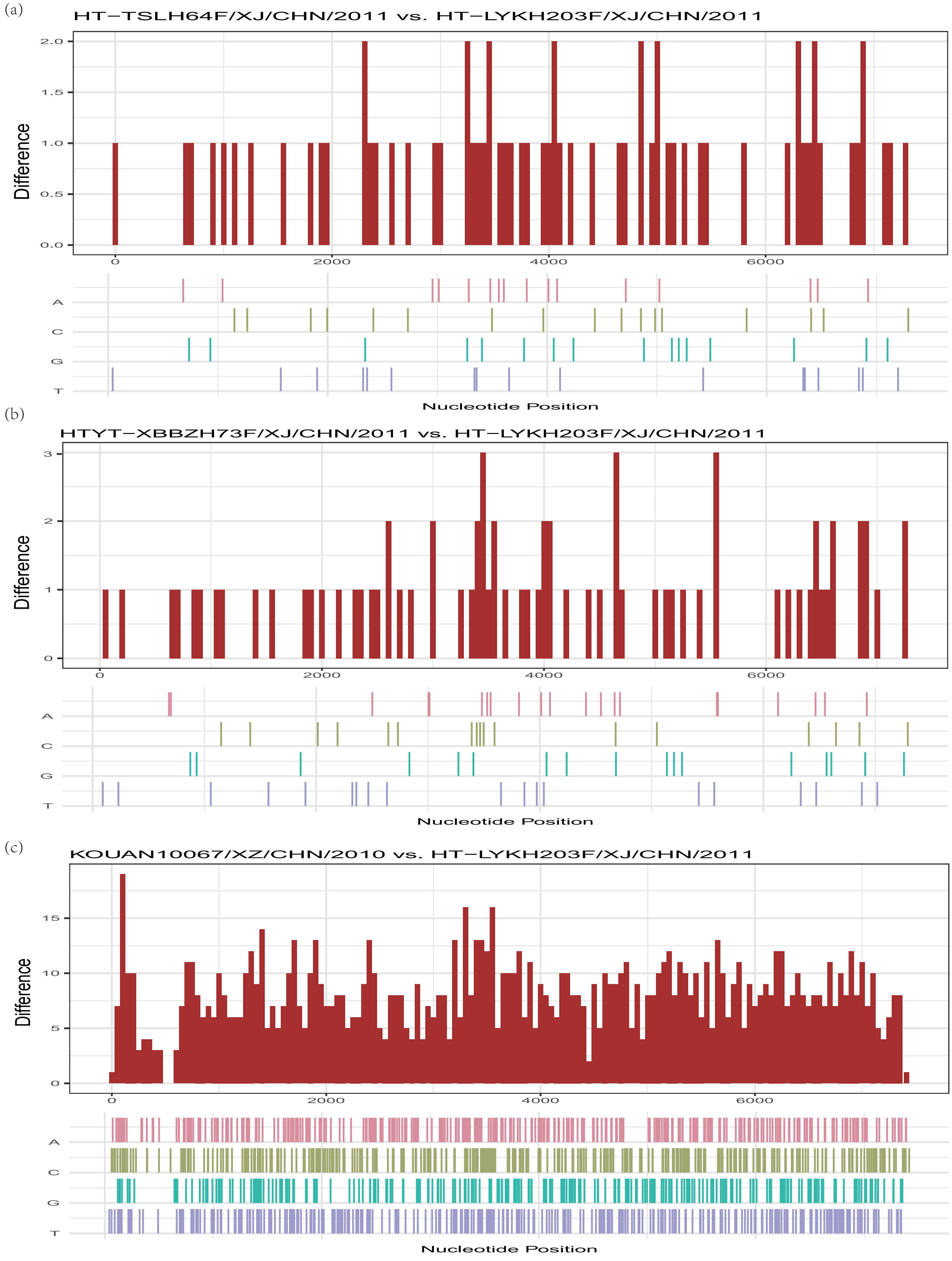

Fig S2. Nucleotide variation across the genome of the three strains(HT-TSLH64F, HTYT-XBB-ZH73F and KOUAN10067) relative to the reference strain(HT-LYKH203F). (a) HT-TSLH64F vs. HT-LYKH203F; (b) HTYT-XBBZH73F vs. HT-LYKH203F; (c) KOUAN10067 vs. HT-LYKH203F.

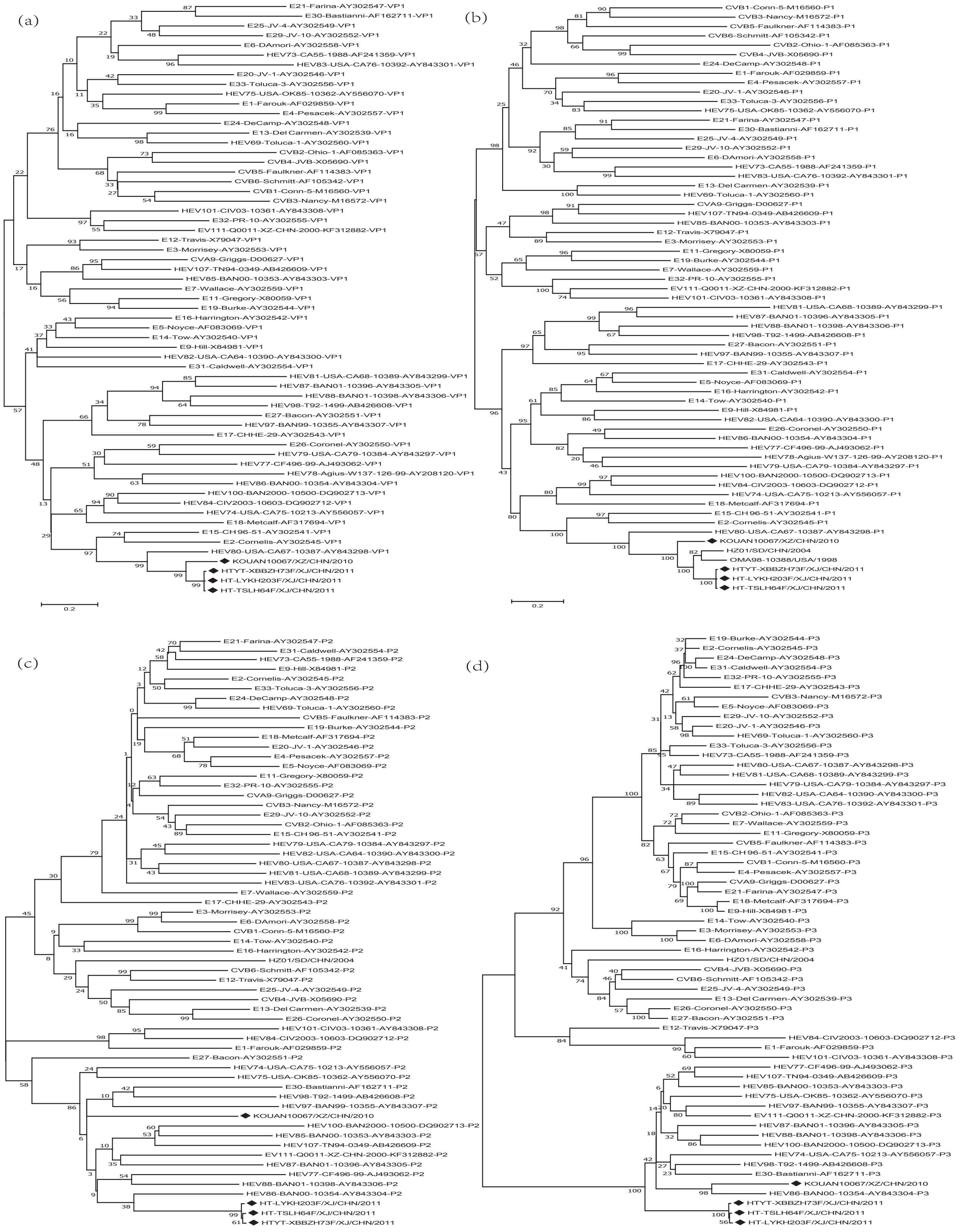

Fig S3. Phylogenetic relationships based the VP1, P1, P2 and P3 coding regions of EV-B. The four EV-B80 strains in this study are indicated by solid diamonds. 57 other EV-B prototypes were analyzed by nucleotide alignment using the Maximum Likelihood method implemented in the MEGA7.0 program. Numbers at the nodes indicate bootstrap support for the node (percentage of 1000 bootstrap replicates). The scale bars represent the substitutions per site per year. (a)VP1 coding sequences; (b)P1 coding sequences; (c)P2 coding sequences; (d)P3 coding sequences.
